# A Single Dose of Modified Vaccinia Ankara expressing Ebola Virus Like Particles Protects Nonhuman Primates from Lethal Ebola Virus Challenge

**DOI:** 10.1038/s41598-017-19041-y

**Published:** 2018-01-16

**Authors:** Arban Domi, Friederike Feldmann, Rahul Basu, Nathanael McCurley, Kyle Shifflett, Jackson Emanuel, Michael S. Hellerstein, Farshad Guirakhoo, Chiara Orlandi, Robin Flinko, George K. Lewis, Patrick W. Hanley, Heinz Feldmann, Harriet L. Robinson, Andrea Marzi

**Affiliations:** 1 GeoVax Inc, Atlanta, GA USA; 2 Rocky Mountain Veterinary Branch, Division of Intramural Research, National Institute of Allergy and Infectious Diseases, National Institutes of Health, Hamilton, MT USA; 3 Laboratory of Virology, Division of Intramural Research, National Institute of Allergy and Infectious Diseases, National Institutes of Health, Hamilton, MT USA; 4 Division of Vaccine Research, Institute of Human Virology, University of Maryland School of Medicine, Baltimore, MD USA

## Abstract

Ebola virus (EBOV), isolate Makona, was the causative agent of the West African epidemic devastating predominantly Guinea, Liberia and Sierra Leone from 2013–2016. While several experimental vaccine and treatment approaches have been accelerated through human clinical trials, there is still no approved countermeasure available against this disease. Here, we report the construction and preclinical efficacy testing of a novel recombinant modified vaccinia Ankara (MVA)-based vaccine expressing the EBOV-Makona glycoprotein GP and matrix protein VP40 (MVA-EBOV). GP and VP40 form EBOV-like particles and elicit protective immune responses. In this study, we report 100% protection against lethal EBOV infection in guinea pigs after prime/boost vaccination with MVA-EBOV. Furthermore, this MVA-EBOV protected macaques from lethal disease after a single dose or prime/boost vaccination. The vaccine elicited a variety of antibody responses to both antigens, including neutralizing antibodies and antibodies with antibody-dependent cellular cytotoxic activity specific for GP. This is the first report that a replication-deficient MVA vector can confer full protection against lethal EBOV challenge after a single dose vaccination in macaques.

## Introduction

Ebola hemorrhagic fever (EHF), now also known as Ebola virus disease, is a fast-progressing, highly lethal zoonosis that, if not contained, poses a threat to global public health. At least 17 outbreaks of EHF have occurred since 1976^1,2^ with the 2013–16 epidemic in West Africa being the first with devastating consequences of rural to urban spread^3^, and the first to cause more than 11,000 fatalities^4^. Given the increasing urbanization of Africa and the globalization of air traffic, measures need to be put in place to control the next emergence of Ebola virus (EBOV). Among countermeasures to EBOV, such as quarantine or supportive and specific treatment, an ideal measure could also include a single-shot vaccine, capable of rapidly providing protective immunity. Over the last decades several experimental EBOV vaccines have been developed^5^, and a few candidates have been accerlated through human clinical trials performed towards the end or after the EBOV epidemic in West Africa. Among them are the fast-acting, single dose live-attenuated vesicular stomatitis virus-based vaccine (rVSV-ZEBOV), and the non-replicating chimpanzee adenodvirus-based vaccine (ChAd3-ZEBOV) in combination with a modified vaccinia Ankara (MVA)-BN-filo boost^5^. While the rVSV-ZEBOV in particular has shown efficacy in a phase III clinical trial in Guinea^6,7^, it is also associated with mild to moderate adverse effects including polyarthritis in a dose-dependent manner^8–10^. Unfortunately, for the ChAd3-ZEBOV/MVA-BN-filo combination no efficacy data are available, only studies have been published associating this vaccine approach with immunogenicity and only mild adverse effects^11,12^. Another approach with promising pre-clinical efficacay data are virus-like particle-based vaccines (VLPs) consisting of the EBOV matrix protein VP40 and the glycoprotein (GP)^5^. This vaccine presents the antigens to the immune system in particles closely resembling authentic EBOV and elicits protective humoral and adaptive immune respones^5^. However, multiple doses of the adjuvanted VLP vaccine are needed to achieve complete protection. Based on existing knowledge and data, we set out to generate a single-dose, non-replicating vaccine capable of forming EBOV-like particles (VLPs). We also replaced the antigens with those from the EBOV-Makona strain to address the evolution of the pathogen.

The decision to use MVA as an EBOV vaccine platform was, in part, based on its parent, vaccinia virus, which has been successfully used in ring vaccinations to contain local outbreaks during the eradication of smallpox^13^. In addition, MVA has been shown to rapidly protect against a lethal monkeypox challenge within 4 days of vaccination^14^. This protection was quicker than could be achieved with the replication competent smallpox vaccine (DryVax), presumably due to the administration of a higher dose of MVA (1 × 10^8^ TCID_50_) which was safely tolerated in humans^15–17^. MVA and MVA-vectored vaccines also afford a high safety profile, primarily by being replication-defective in humans as they can only replicate in avian cells, in which they are propagated^18,19^. Due to its large genetic size, and its loss of genes during attenuation, MVA has the capacity to readily express more than one transgene, facilitating the expression of sufficient foreign viral proteins for the formation of VLPs. Finally, vaccinia virus elicits durable antibody (Ab) responses^20^, a phenomenon that appears to be conferred on immune responses to transgenes expressed by recombinant MVA vaccines. Taken together, the MVA-EBOV vaccine could be very useful - not only for containing an outbreak by emergency immunization, but as a routine vaccine for a target population in EBOV endemic areas.

In this study, we report the construction of a MVA-based vaccine designed to express the EBOV GP on the surface of non-infectious VLPs. The VLPs are formed by the self-assembly and budding of MVA-expressed EBOV matrix protein VP40 with EBOV GP, also encoded by MVA, presented on their surface^21–23^. GP is the main target for protective Ab responses^24,25^, and its display on the VLP surface efficiently initiates Ab and T cell responses similar to EBOV infection. Here, we analyzed the protective efficacy of this novel recombinant MVA-based EBOV vaccine in two well-established animal disease models for EBOV infection, the guinea pig and macaques.

## Results

### Vaccine construction

**Figure 1:**
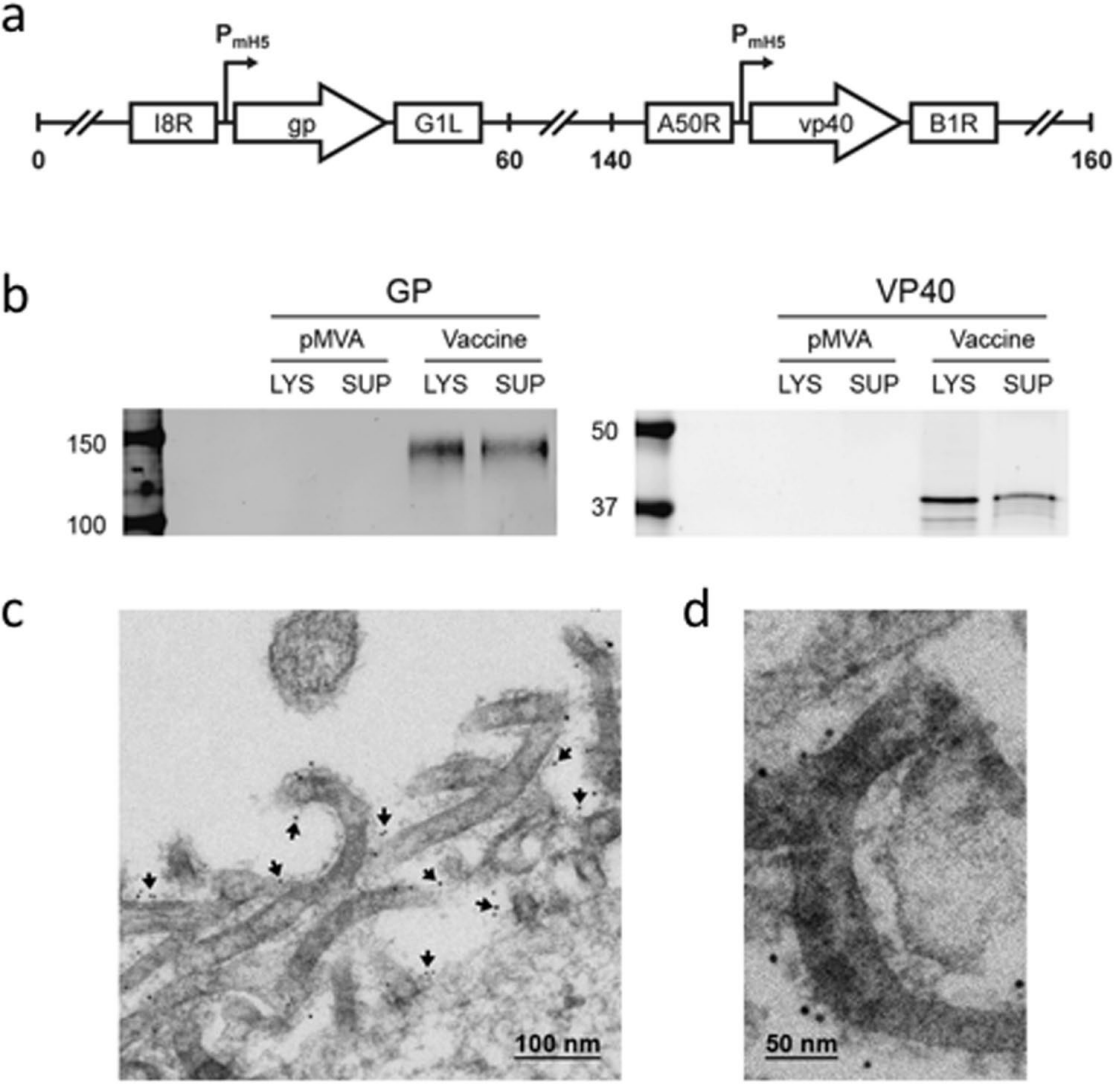
MVA-EBOV vaccine construction and expression. (**a**) Schematic of MVA-EBOV vaccine expressing EBOV-like particles. I8R and G1L, conserved vaccinia sequences flanking the insertion site for *gp;* A50R and B1R, conserved sequences flanking the insertion site for *vp40*. P_mH5_, modified early late H5 vaccinia promoter. Numbers indicate positions in the MVA genome which is abbreviated and not to scale. (**b**) Western blot showing expression of EBOV GP (monoclonal Ab c6D8) and VP40 (polyclonal Ab) in 293 T cell lysates (LYS) and supernatants (SUP). Molecular weight markers are indicated to the left of blots. (**c**) Thin section electron micrograph showing the expression of EBOV-like particles. Arrows in (**c**) show examples of immunogold staining for GP. (**d**) Magnified thin section electron micrograph showing the immunogold staining of GP (black dots) without arrows.

A recombinant MVA vector was generated expressing the EBOV GP and VP40 antigens based on the sequence of an EBOV-Makona 2014 isolate (GenBank Accession Number KM233103), the causative agent of the 2013–16 West African epidemic (Fig. 1a). This vector, designated MVA-EBOV, expresses GP and VP40 in vaccine-infected cells and VLPs are released from these cells. Western blot analysis revealed correctly sized bands confirming the expression of GP and VP40 in the lysates and supernatants of infected 293 T cells (Fig. 1b). Thin section electron microscopy documented the active budding of VLPs from these cells, and immuno-gold staining confirmed the display of EBOV GP on the surface of the VLPs (Fig. 1c,d).

### Vaccine efficacy testing in guinea pigs

**Figure 2:**
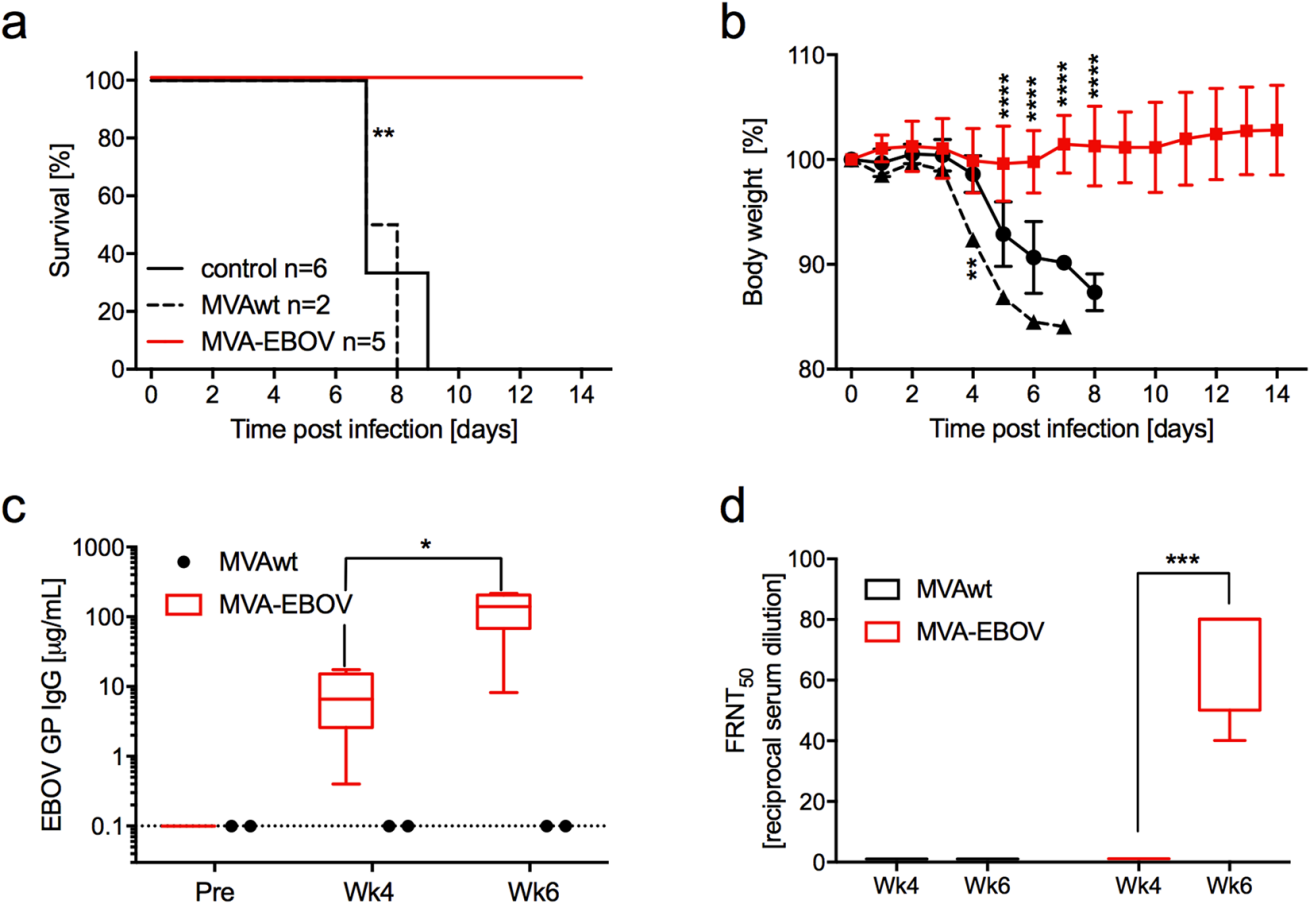
MVA-EBOV protection study in guinea pigs. (**a**) Survival curves for animals primed at week 0 and boosted at week 4 with MVA-EBOV or MVAwt. Controls are unvaccinated animals. At 8 weeks (day 0) all animals were challenged with the EBOV-Mayinga-based GPA-EBOV. (**b**) Body weight changes post challenge. (**c**) Anti-GP-specific Ab measured pre-immunization (pre), at 4 weeks after the prime (Wk4) and 2 weeks after the boost (Wk6). (**d**) Neutralizing Ab measured as 50% focus reduction neutralization titer (FRNT_50_). Significance levels are indicated as follows: p < 0.05 (*), p < 0.01 (**), p < 0.001 (***) and p < 0.0001 (****).

The protective potential of this new MVA-EBOV vaccine was evaluated in an established rodent model for EBOV, the guinea pig, using the EBOV-Mayinga-based guinea pig-adapted strain (GPA-EBOV). Young adult guinea pigs were prime and boost vaccinated with 10^8^ TCID_50_ of MVA-EBOV at week 0 (prime) and week 4 (boost) and were challenged at week 8 with a lethal dose of GPA-EBOV. While the guinea pigs in the unvaccinated and the MVAwt vaccinated control groups developed signs of disease and were euthanized after reaching the humane endpoint, the MVA-EBOV vaccinated guinea pigs showed little to no body weight changes after challenge and survived (Fig. 2a,b). Estimated median values of EBOV GP-specific IgG of 6.6 µg/ml at 4 weeks post prime and 140 µg/ml at 2 weeks post boost (week 6)(Fig. 2c) demonstrated good immunogenicity of the MVA-EBOV delivered antigen. Neutralizing Ab scored with a median 50% focus reduction neutralization titer (FRNT_50_) that was below detection at 4 weeks post prime, but at 1:80 at 2 weeks post boost (week 6)(Fig. 2d).

### Vaccine efficacy testing in macaques

Given the achievement of 100% protection in the guinea pig model, the efficacy of MVA-EBOV was next tested in macaques against lethal challenge with EBOV-Makona. Twelve rhesus macaques were divided into 3 groups of 4 animals resulting in a control group (MVAwt), a MVA-EBOV prime/boost and a MVA-EBOV prime group. The first dose of 10^8^ TCID_50_ of MVA-EBOV for the prime/boost regimen was given intramuscularly (IM) on day −56 followed by the boost on day −28. For the prime regimen, the single dose vaccination of 10^8^ TCID_50_ of MVA-EBOV was delivered on day −28. Unvaccinated animals received two IM injections with the same dose of MVAwt following the prime/boost regimen. Challenge was by IM injection of 1,000 plaque-forming units of EBOV-Makona on day 0.

**Figure 3:**
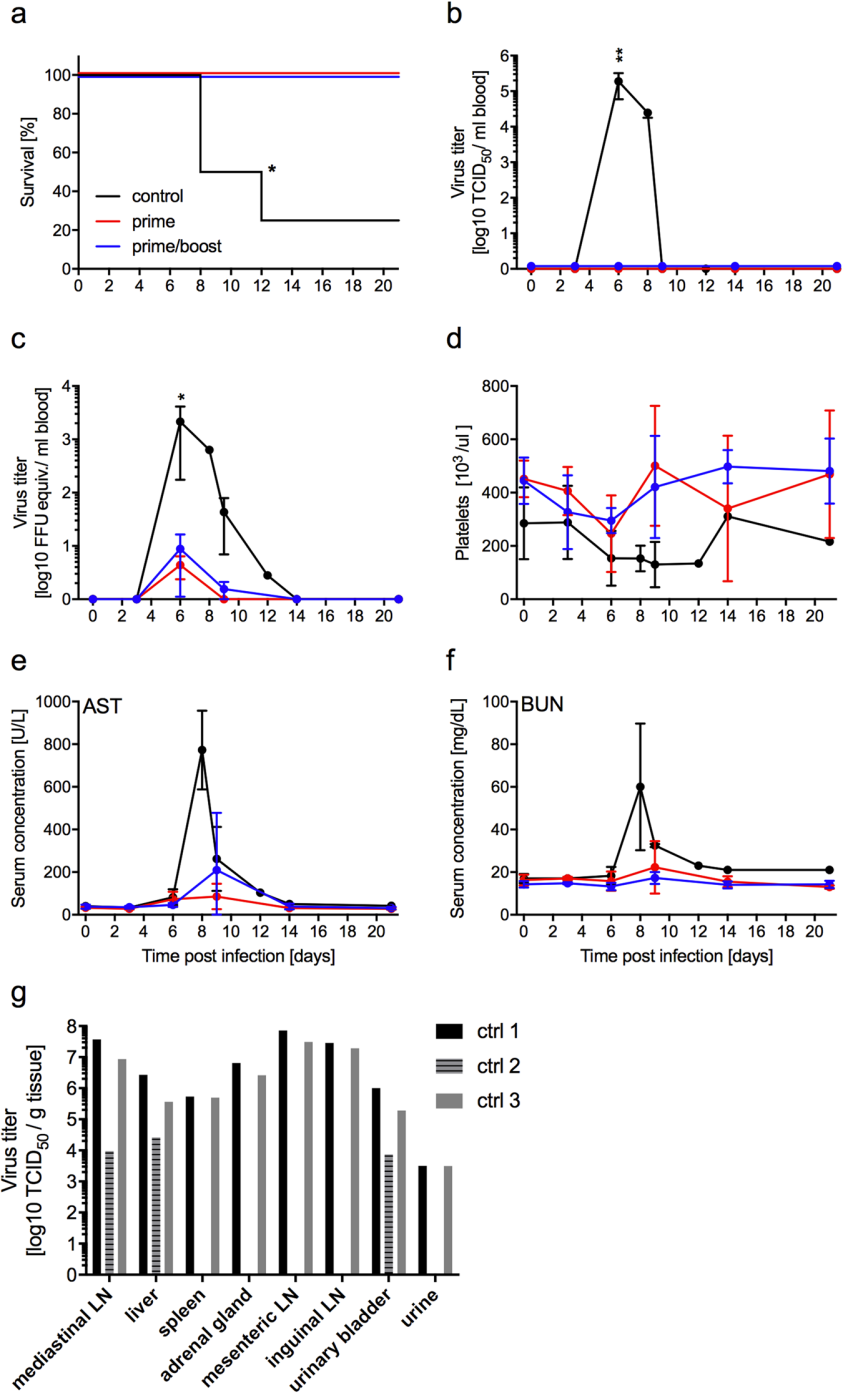
MVA-EBOV protection study in rhesus macaques. (**a**) Survival curves of animals receiving prime or prime/boost with MVA-EBOV or MVAwt (control) and challenge with EBOV-Makona on day 0. Viremia levels determined in whole blood samples over time by (**b**) virus titration and (**c**) RT-qPCR. (**d**) Counts of platelets in macaque whole blood samples over time. Levels of (**e**) aspartate amino transferase (AST) and (**f**) blood urea nitrogen (BUN) in the serum of challenged macaques over time. (**g**) EBOV titers in selected tissue samples of MVAwt control animals at the time of euthanasia. Significance levels are indicated as follows: p < 0.05 (*), and p < 0.01 (**). All other results are not statistically significant.

Both immunization schemes, prime as well as prime/boost vaccination, resulted in 100% protection from lethal EHF in the animals (Fig. 3a). In contrast, all the animals in the MVAwt control group developed EHF with three animals reaching euthanasia criteria and one animal recovering (Fig. 3a). Only the control animals developed EBOV viremia at 6 days after challenge and the time of euthanasia as measured by virus isolation from the blood (Fig. 3b). However, all 8 vaccinated animals developed low-level viremia as detected by RT-qPCR (Fig. 3c). The one control that survived developed only mild signs of disease, and while viremia was detected using RT-qPCR, virus was never isolated from its blood. Platelets underwent a transient decrease in the vaccinated animals but a more pronounced and prolonged decrease in the MVAwt control animals, including the one MVAwt animal that survived (Fig. 3d). The lowest point for platelet counts was on day 6 post challenge for both MVA-EBOV vaccinated groups (Fig. 3d). Vaccination with MVA-EBOV limited the development of EHF as documented by measurable but limited increases of aspartate amino transferase (AST) (Fig. 3e) and blood urea nitrogen (BUN) (Fig. 3f) in serum on day 9 post challenge. For both the hematology and blood chemistry measurements, the prime group demonstrated slightly lower values than the prime/boost group suggesting that priming had provided comparable protection to priming and boosting. As expected and described previously^26^, the control animals that succumbed to infection had high EBOV titers in selected tissues such as lymph nodes, spleen, adrenal gland, urinary bladder and urine (Fig. 3g). Interestingly, the control animal that succumbed on day 12 after challenge had decreased levels of virus, and EBOV was only isolated from a few of the selected tissues (Fig. 3g; ctrl 2).

**Figure 4:**
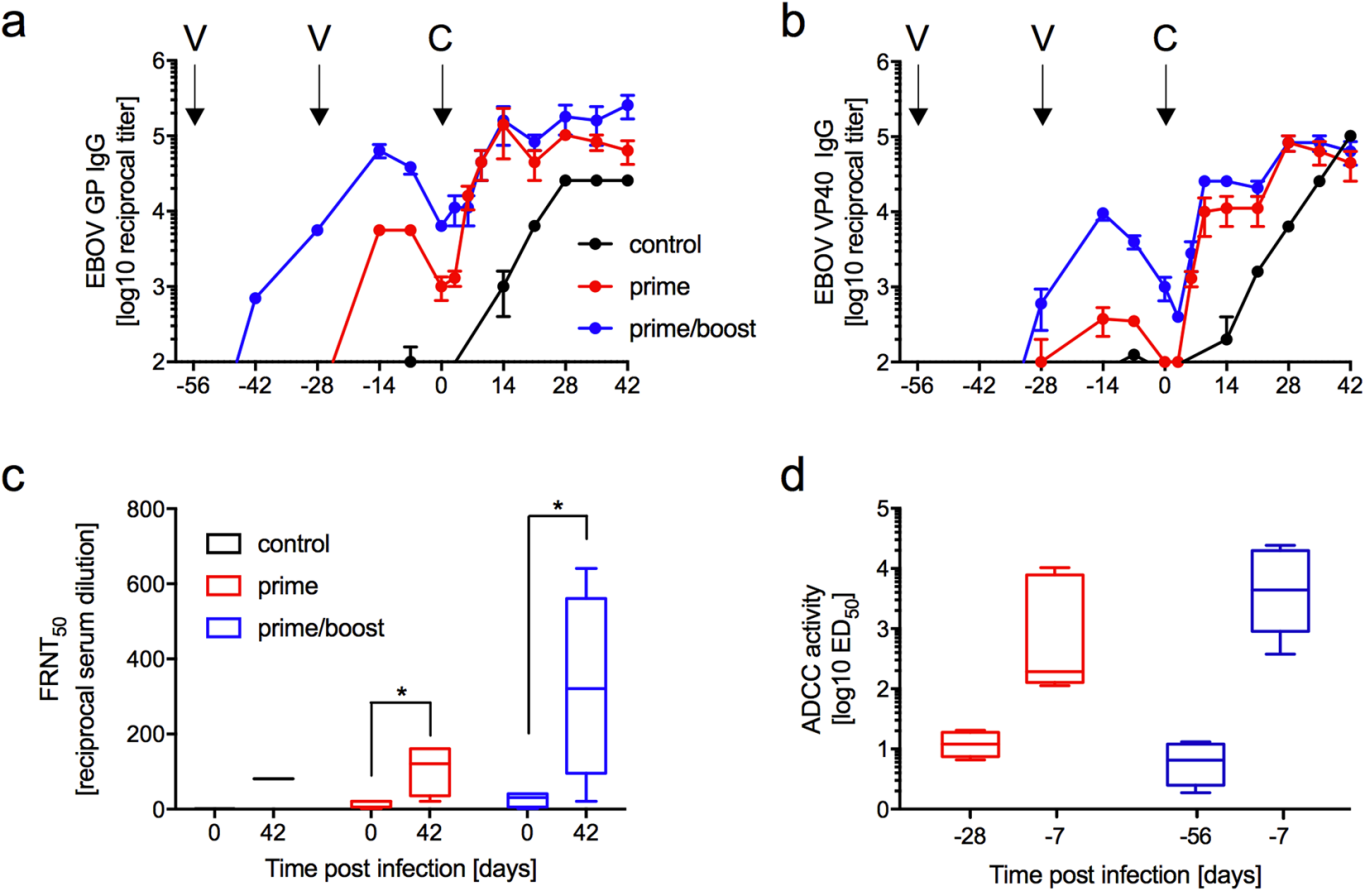
Humoral responses after vaccination and challenge. (**a**) EBOV GP-specific and (**b**) EBOV VP40-specific IgG during the immunization and challenge phases of the study. Arrows indicate days of vaccination (V; day −56, −28) and challenge (C, day 0). (**c**) Neutralizing titers on day 0 and 42 of the study. (**d**) ADCC activity pre-immunization (day −28 for prime group and day −56 for prime/boost group) and on day −7 (pre-challenge). Significance level is indicated as p < 0.05 (*); all other results are not statistically significant.

The MVA-EBOV vaccine elicited IgG responses specific to both EBOV GP and VP40 as determined by ELISA (Fig. 4a,b). On the day of challenge, the IgG levels were 16-times higher (average titer of 1:6,400) for GP and 8-times higher for VP40 (average titer of 1:3,200) in the prime/boost group compared to the prime group. Despite this measurable but not significant difference in Ab titers, the animals in both groups showed comparable signs of disease and levels of viremia as detected by RT-qPCR (Fig. 3c). In both groups, the challenge stimulated strong anamnestic GP- as well as VP40-specific responses. On day 6 post challenge, both groups had similar levels of total GP-specific IgG (Fig. 4a), whereas VP40-specific IgG was slightly higher in the prime/boost group until day 21 post challenge (Fig. 4b). Sera collected from all of the vaccinated animals on the day of challenge (day 0) had neutralizing activity (up to 1:40) as measured in a FRNT_50_ assay against the challenge virus EBOV-Makona (Fig. 4c). As expected and described previously for other vaccines^27^, neutralizing titers increased after challenge in the surviving animals to a titer up to 1:160 for the prime and a titer up to 1:640 for the prime/boost group (Fig. 4c).

In addition to neutralization, another possible protective function for vaccine-induced Ab is antibody-dependent cell-mediated cytotoxicity (ADCC). To gain further insight into the functionalities of the Ab elicited by the MVA-EBOV vaccine, we performed ADCC assays using cells expressing the EBOV-Kikwit GP. The ADCC assay revealed median titers measured as effective dose 50 (ED_50_) of 1:193 for the prime group, and 1:4, 391 for the prime/boost group at 7 days prior to challenge (Fig. 4d).

## Discussion

In this study, we demonstrated the ability of a new MVA-EBOV vaccine to provide single dose protection against a lethal EBOV-Makona challenge in macaques. This vaccine expresses the EBOV-Makona antigens GP and VP40 and, therefore, EBOV-like particles are released from vaccine-infected cells (Fig. 1). This is the first study reporting protective efficacy in rodents and NHPs of any MVA-EBOV vaccine expressing EBOV-like particles. In addition, this is one of only two studies successfully testing vaccines expressing EBOV-Makona antigens in a homologous challenge setting in NHPs^28^. EBOV-like particle production by a vaccine has previously been described for vesicular stomatitis virus (VSV)-EBOV vaccination^29^. Recently, an MVA-BN-EBOV vaccine also forming VLPs was described by Schweneker *et al*.^30^. This MVA vaccine expresses GP and VP40 from the EBOV-Mayinga strain in addition to the *Tai Forest ebolavirus* nucleoprotein NP. While the addition of NP *in vitro* enhances EBOV-like particle production^23^, the inclusion of NP into other platforms like the rVSV- or rAd5- based EBOV vaccines has resulted in decreased protective efficacy in rodent and NHP models^29,31^. Therefore, we did not include NP in our vaccine.

In Guinea pigs, prime/boost vaccination with the MVA-EBOV elicited adequate humoral immune responses as documented by total EBOV GP-specific Ab and neutralizing titers, and all the vaccinated animals were protected against the lethal GPA-EBOV challenge (EBOV-Mayinga-based strain)(Fig. 2c,d).

The demonstration of protective responses in guinea pigs warranted efficacy testing in macaques, the closest model for EBOV-induced human disease used in countermeasure development. A comparison between a prime/boost and a single-dose (prime only) immunization against homologous EBOV-Makona challenge resulted in 100% protection for both vaccination groups. Given the ability of MVA to rapidly protect against monkey poxvirus infections^14^ and the ability of a single dose of MVA-EBOV to achieve protection against EBOV in NHPs, MVA-EBOV could be an effective emergency vaccine similar to rVSV-ZEBOV. In clinical trials during the 2013–2016 West African epidemic, the replication competent rVSV-ZEBOV was associated with no contact infections 10 days after vaccination in Guinea^6^, suggesting that the vaccine could prevent infections within 10 days of administration. In cynomolgus macaques this same vaccine offered complete protection when administered 7 days prior to lethal EBOV-Makona challenge and partial protection for those animals vaccinated 3 days prior to challenge^27^. Given the immune responses obtained during this study are comparable to those elicited by rVSV-ZEBOV in macaques, this MVA-EBOV vaccine would provide a safer single-dose alternative as it is not associated with transient side effects like oligoarthritis^8–10^. A single dose MVA-EBOV vaccine is also easier to manufacture and deploy than recombinant adenovirus, subunit/protein, DNA and other MVA vaccines that require multiple doses, or a heterologous prime/boost protocol for efficacy.

In contrast to MVA-EBOV, which holds promise in these initial preclinical studies for being an effective single dose vaccine, a heterologous adenovirus prime/MVA boost regimen is being used in the development of MVA-BN-filo, a MVA filovirus vaccine co-expressing the GPs of EBOV, Sudan virus and Marburg virus^11,32,33^. MVA vaccines expressing multiple filovirus GPs have not protected as single dose vaccines against lethal EBOV challenge^34^. We hypothesize that the success of our single dose MVA-EBOV vaccine reflects the efficient initiation of B cell responses by VLPs displaying the GP.

The one surviving animal in the MVAwt vaccinated control group neither reached a high clinical score nor developed concerning signs of disease. This is not the first macaque described to survive EBOV-Makona challenge, which has been observed by us and others in macaques that received various EBOV-non-specific treatments after challenge^26,35^. Thus, it appears that in rhesus macaques, the development of lethal EHF after EBOV-Makona challenge is not as uniform compared to other challenge virus isolates.

Because of uniform protection, we could not assess a correlate for protection. However, we consider it likely that good levels of neutralizing Ab plus good levels of ADCC, a non-neutralizing Ab activity that kills infected cells, contributed to protection.

For now, priorities for the continued development of this MVA-EBOV-like particle vaccine include testing the rapidity for onset of protection and the durability of the protective responses.

## Methods

### Animal ethics and biosafety statement

All guinea pig and macaque work was performed in strict accordance with the recommendations described in the Guide for the Care and Use of Laboratory Animals of the National Institute of Health, the Office of Animal Welfare and the United States Department of Agriculture. Animal procedures were carried out under anesthesia by trained personnel under the supervision of veterinary staff and all efforts were made to promote the welfare and to minimize the suffering of animals. The macaque study was performed in accordance with the recommendations of the “Weatherall report for the use of non-human primates”.

Guinea pig vaccinations were carried out at Bioqual Inc (Rockville, MD) and were approved by the Institutional Animal Care and Use Committee (IACUC). All animal work with infectious EBOV was performed in the maximum containment laboratory at the Rocky Mountain Laboratories (RML), Division of Intramural Research (DIR), National Institute of Allergy and Infectious Diseases (NIAID), National Institutes of Health (NIH), Montana, USA applying standard operating protocols approved by the Institutional Biosafety Committee (IBC). Guinea pig and macaque studies were approved by the RML IACUC. Guinea pigs were housed in approved isolator cages under controlled conditions of humidity, temperature and light (12-hour light/12-hour dark cycles). Macaques were housed in adjoining individual primate cages allowing social interactions, under controlled conditions of humidity, temperature and light (12-hour light/12-hour dark cycles). Food and water were available *ad libitum*. Animals were monitored at least twice daily (pre- and post-infection) and fed commercial chow, treats and fruit twice daily by trained personnel. Macaque environmental enrichment consisted of commercial toys, video and music. Humane endpoint criteria, specified and approved by the RML IACUC, were applied to determine when animals should be humanely euthanized.

### Vaccine construction and expression testing

Vaccine constructions were done with sequences from the 2014 EBOV-Makona (GenBank Accession number KM233103), a strain representative of the 2013–2016 West African epidemic. We used a parental MVA that had been harvested in 1974 before the appearance of bovine spongiform encephalopathy (BSE) and sent in 2001 to Dr. Bernard Moss at NIAID, where it was plaque purified 3 times using certified reagents from sources free of BSE. The pLW76 shuttle vector (Wyatt and Moss, unpublished data) was used to place VP40 sequences in a modified and restructured insertion site III and the pLW73 shuttle vector to insert GP sequences between two essential vaccinia genes (I8R and G1L)(Fig. 1a). VP40 and GP inserts were optimized for the codon usage of vaccinia virus and sequences encoding termination of vaccinia transcripts were eliminated by using alternate codons. The shuttle vectors used the modified H5 early/late promoter to drive transcription^36^. Western blot analysis was conducted on supernatants and lysates of 293 T cells 48 hours after infection using standard practices. The murine-human chimeric monoclonal Ab c6D8 (IBT) was used as the primary Ab for GP and a polyclonal rabbit Ab as the primary Ab for VP40 (IBT). Immuno-electron microscopy was conducted on 48 hour infected DF-1 cells as previously described only using the c6D8 monoclonal as the primary Ab for GP. Immuno-gold staining was performed with goat anti-human coated 6 nm colloidal gold particles.

### Challenge viruses

Guinea pig-adapted (GPA-) EBOV (based on EBOV-Mayinga)^29^ and EBOV-Makona (Guinea C07; passage 1)^26,27^ were propagated on Vero E6 cells (mycoplasma negative), titrated on these cells and stored in liquid nitrogen. Deep sequencing confirmed that the prevailing phenotype for both EBOV stocks was 7U.

### Guinea pig study

Six 3–4 weeks old female Hartley guinea pigs were inoculated intramuscularly (IM) with 1 × 10^8^ TCID_50_ of MVA-EBOV at week 0 and week 4; similarly, 2 guinea pigs received the same dose of MVAwt at the same time. Sera were collected at week 0 (pre-immunization), week 4 (day of boost) and at week 6 (2 weeks after the boost). The vaccinated guinea pigs as well as 6 additional weight and age matched naive control animals were then shipped to the RML for challenge. On the day of challenge all guinea pigs were infected intraperitoneally with 10 focus-forming units (=1,000 LD_50_) of GPA-EBOV and were weighed and monitored daily for signs of disease.

### Macaque study

A total of 12 rhesus macaques (*Macaca mulatta*), 3 female and 9 male animals, 4–12 years of age and 3.5–8 kg in weight, were used in this study. The macaques were randomly divided into 3 study groups (n = 4, one female and 3 males per group). The animals were immunized IM in the left and the right caudal thigh with 1 × 10^8^ TCID_50_ MVA-EBOV (1 ml inoculum) on days −56 and −28 (prime/boost group) or day −28 (prime only group). The control animals received the same doses of MVAwt via the same route and at the same time as the prime/boost group. All animals were challenged IM on day 0 with a lethal dose of 1,000 PFU EBOV-Makona (1 ml inoculum; confirmed by back-titration) in the left and the right caudal thigh. Physical examinations and blood draws were performed on days −56, −42, −28, −14, −7, 0, 3, 6, 9, 14, 21, 28, 35 and 42 as well as at the time of euthanasia. The animals were observed at least twice daily for clinical signs of disease using an IACUC approved scoring sheet. A score (0–15) was assigned for general appearance, skin and fur, nose/mouth/eyes/head, respiration, feces and urine, food intake, and locomotor activity. These scores were recorded on a daily observation sheet and animals were euthanized by experienced personnel when the total value reached the critical number of 35, or any of the following signs were observed: impaired ambulation preventing access to food or water; excessive weight loss; lack of mental and physical alertness; difficult labored breathing or prolonged inability to remain upright.

### Virus loads

For detection of EBOV-specific RNA in the blood of infected macaques, total RNA was isolated from 140 μl EDTA blood samples on the exam day using the QIAmp viral Mini RNA kit (Qiagen). All quantitative real-time RT-PCRs were performed with the QIAquick 1-step Rotorgene kit (Qiagen) and EBOV polymerase L gene -specific primers as described previously^26^. For titration of macaque blood and tissue samples, Vero E6 cells (mycoplasma negative) were seeded in 48-well plates the day before infection. Blood samples were thawed and 10-fold serial dilutions were prepared. Tissues were homogenized in 1 ml plain DMEM and 10-fold serial dilutions were prepared. Media were removed from cells and triplicates wells were inoculated with each dilution. After one hour, DMEM supplemented with 2% FBS, penicillin/streptomycin and L-glutamine was added and cells were incubated at 37 °C. Cells were monitored for cytopathic effect (CPE) and 50% tissue culture infectious dose (TCID_50_) was calculated for each sample employing the Reed and Muench method.

### Hematology and serum chemistries

The total white blood cell count, lymphocyte, neutrophil, platelet, reticulocyte and red blood cell counts, hemoglobin, and hematocrit values were determined from EDTA blood with the IDEXX ProCyte DX analyzer (IDEXX Laboratories). Serum biochemistry was analyzed using the Piccolo Xpress Chemistry Analyzer and Piccolo General Chemistry 13 Panel discs (Abaxis).

### Enzyme-linked immunosorbent assay (ELISA)

Amounts of EBOV GP-specific IgG in guinea pig sera was determined by ELISAs as previously described with modifications for the specific Ab under test^29^. To this end, ELISA plates were coated with recombinant EBOV-Mayinga GP (IBT Bioservices) and bound Ab was detected with goat anti-guinea pig IgG conjugated to horseradish peroxidase (KPL). OD values were normalized to a standard curve of guinea pig IgG (Jackson ImmunoResearch) and results interpolated to estimate μg GP-specific antibody per milliliter of serum. For rhesus macaque sera, the plates were coated with EBOV-Makona GP (Alpha Diagnostics) or VP40 antigen (produced as described previously^26^) overnight at 4 °C as previously described^27^. After three washes with PBS/0.05% Tween, serial 2-fold or 4-fold dilutions of the serum samples were incubated for 1 h at 37 °C. Following washing three times with PBS/0.05% Tween, horseradish peroxidase (HRP)-conjugated anti-monkey IgG (KPL) was added for 1 h, followed by additional washes and final addition of substrate (KPL). IgG endpoint titers were calculated using log-log transformation of the linear portion of the curve, and 0.1 optical density (OD) units as cut-off. IgG titers were standardized using a positive control sample that was included on every ELISA plate.

### 50% focus reduction neutralization titer (FRNT50)

Neutralizing antibody titers were determined and calculated as FRNT_50_. Briefly, Vero E6 cells (mycoplasma negative) were seeded into 96-well plates to generate a confluent monolayer on the day of infection. Two-fold serum dilutions were prepared in triplicate in plain DMEM and 25 µl were incubated with 200 PFU EBOV-Makona (for rhesus macaque samples) or EBOV-Mayinga (for guinea pig samples) in a total volume of 50 µl for 60 min at 37 °C. Media was removed from cells, the serum-virus mixture was added and samples were incubated for further 60 min at 37 °C. The mixture was removed from the cells and 100 µl of 1.2% carboxymethyl cellulose in MEM (2% FBS) was added per well and left for 4 days at 37 °C. The cells were fixed in 10% neutral buffered formalin and removed from the maximum containment laboratory according to approved standard operating procedures (SOPs). Foci were stained using an anti-EBOV-VP40 polyclonal rabbit serum kindly provided by Yoshihiro Kawaoka (University of Wisconsin-Madison) and a secondary anti-rabbit FITC antibody (Sigma). Foci were counted and the neutralizing activity was determined as percent reduction of EBOV infection compared to control infected cells without serum.

### Antibody-dependent cell-mediated cytotoxicity (ADCC)

The ADCC activity of EBOV GP-specific IgG was quantified with an EBOV-adapted modification of the RFADCC assay described by Orlandi and colleagues^37^. Briefly, a target cell line was made by transfecting 293FS cells with a full-length DNA expressing GP from the EBOV-Kikwit isolate followed by transfecting with two separate DNA constructs expressing EGFP and the chimeric CCR5-SNAP tag protein. The new cell line, designated EBOV GPkik-293FS EGFP CCR5-SNAP, expresses EBOV-Kikwit GP on the plasma membrane, EGFP in the cytoplasm and the SNAP-Tag CCR5, which can be specifically labeled with SNAP-Surface Alexa Fluor-647 (NEB), on the cell surface (*manuscript in preparation*). A mixture of three anti-EBOV GP chimeric antibodies (c6D8, c13C6 FR1 and 13F6) (IBT) and the human anti-EBOV antibody KZ52 (a neutralizing antibody)(IBT) were used as positive controls and the unrelated human antibody Synagis as a negative control. The ADCC activity was quantified by incubating three-fold serial dilutions of anti-EBOV antibodies with EBOV GPkik-293FS EGFP CCR5-SNAP target cells for 15 mins at RT and then adding human PBMC as effector cells for 2 hours at 37°, after which cells were washed once with PBS, fixed with 2% paraformaldehyde, stained and analyzed with an LSRII Fortessa flow cytometer (BDBiosciences). Data analysis was performed with FlowJo software (Tree Star,Inc., San Carlos, Calif.). The percentage cytotoxicity of the anti-EBOV antibodies was determined as the number of target cells losing EGFP (by virtue of ADCC) but retaining the surface expression of CCR5 SNAP.

### Statistical analyses

All statistical analysis was performed in Prism 7 (GraphPad). All survival curves were compared with the Kaplan Meier survival test. For each time point, all groups were tested using Two-Way ANOVA with Tukey’s multiple comparisons.
